# Ovarian dysgerminomas are characterised by frequent *KIT *mutations and abundant expression of pluripotency markers

**DOI:** 10.1186/1476-4598-6-12

**Published:** 2007-02-02

**Authors:** Christina E Hoei-Hansen, Sigrid M Kraggerud, Vera M Abeler, Janne Kærn, Ewa Rajpert-De Meyts, Ragnhild A Lothe

**Affiliations:** 1 University Dept. of Growth & Reproduction, Rigshospitalet, Copenhagen, Denmark; 2 Dept. of Cancer Prevention, Institute for Cancer Research, The Rikshospitalet-Radiumhospitalet Medical Centre, Oslo, Norway; 3 Dept. of Pathology, The Rikshospitalet-Radiumhospitalet Medical Centre, Oslo, Norway; 4 Dept. of Gynecologic Oncology, The Rikshospitalet-Radiumhospitalet Medical Centre, Oslo, Norway; 5 Center for Cancer Biomedicine, University of Oslo, Norway

## Abstract

**Background:**

Ovarian germ cell tumours (OGCTs) typically arise in young females and their pathogenesis remains poorly understood. We investigated the origin of malignant OGCTs and underlying molecular events in the development of the various histological subtypes of this neoplasia.

**Results:**

We examined *in situ *expression of stem cell-related (NANOG, OCT-3/4, KIT, AP-2γ) and germ cell-specific proteins (MAGE-A4, NY-ESO-1, TSPY) using a tissue microarray consisting of 60 OGCT tissue samples and eight ovarian small cell carcinoma samples. Developmental pattern of expression of NANOG, TSPY, NY-ESO-1 and MAGE-A4 was determined in foetal ovaries (gestational weeks 13–40). The molecular genetic part of our study included search for the presence of Y-chromosome material by fluorescence *in situ *hybridisation (FISH), and mutational analysis of the *KIT *oncogene (exon 17, codon 816), which is often mutated in testicular GCTs, in a subset of tumour DNA samples. We detected a high expression of transcription factors related to the embryonic stem cell-like pluripotency and undifferentiated state in OGCTs, but not in small cell carcinomas, supporting the view that the latter do not arise from a germ cell progenitor. Bilateral OGCTs expressed more stem cell markers than unilateral cases. However, *KIT *was mutated in 5/13 unilateral dysgerminomas, whereas all bilateral dysgerminomas (n = 4) and all other histological types (n = 22) showed a wild type sequence. Furthermore, tissue from five phenotypic female patients harbouring combined dysgerminoma/gonadoblastoma expressed TSPY and contained Y-chromosome material as confirmed by FISH.

**Conclusion:**

This study provides new data supporting two distinct but overlapping pathways in OGCT development; one involving spontaneous *KIT *mutation(s) leading to increased survival and proliferation of undifferentiated oogonia, the other related to presence of Y chromosome material and ensuing gonadal dysgenesis in phenotypic females.

## Background

The three main categories of ovarian tumours are: surface epithelium-stromal tumours, sex-cord tumours and germ cell tumours (GCTs; benign and malignant). Malignant ovarian (OGCTs) have a median age at onset of 18 years and represent approximately 3% of all ovarian cancers in Western countries [1]. The following histological sub-types exist: dysgerminoma, yolk sac tumour, embryonal carcinoma, polyembryoma, choriocarcinoma, immature teratoma, and mixed GCTs. Bilateral tumours occur in up to 10% of cases [1,2]. The correct differential diagnosis is imperative since the prognosis and choice of therapy remain different among the various ovarian cancer types. A treatment consisting of a combination of surgical resection and platinum-based chemotherapy cures the majority of malignant OGCT patients [2]. The fact that OGCTs often affect women in their reproductive years further imply the importance of optimal therapy in order to maximize the number of women in which ovarian function can be conserved.

Many similarities exist between GCTs of the ovary and testis, including a morphological resemblance and a similar pattern of chromosomal alterations [3,4]. Furthermore, families with both ovarian and testicular GCTs have been reported, suggesting a possible association/common genetic aetiology [5,6]. Ovaries and testes develop similarly until approximately 2 months of embryonic life, which is also consistent with a common origin of, at least some cases of, ovarian and testicular GCTs. In this study, recent knowledge of underlying mechanisms in development of testicular GCTs was used as a guide to investigate patterns of *in situ *protein expression in OGCTs. As a close resemblance between testicular GCTs and embryonic stem cells has been shown [7] particular focus was on stem cell-related factors including KIT (also known as c-Kit, tyrosine kinase receptor for stem cell factor (SCF)), OCT-3/4 (POU5F1, a POU-family transcription factor), NANOG, and AP-2γ (TFAP2C, transcription factor activator protein-2), and focus was also on germ cell-specific proteins (including MAGE-A4 and NY-ESO-1 belonging to the cancer/testis gene family) with a cell differentiation related biological function or a developmentally regulated expression pattern [8]. KIT is involved in the migration of primordial germ cells (PGCs) [9] and there has been reported a frequent presence of *KIT *mutations in GCTs and in particular bilateral testicular GCTs [10-17]. Therefore *KIT *mutation status was also determined in the same samples and we established the expression pattern of proteins not previously studied in the ovary during foetal ovarian development. Finally, patients with intersex disorders and dysgenetic ovaries have an increased risk of harbouring a GCT, and the ovarian malignancies were therefore investigated for the presence of Y-chromosome material. Gonadoblastomas are rare neoplasms composed of germ cells and immature granulosa/Sertoli cells that develop nearly exclusively in males and phenotypic females harbouring Y-chromosome material. We chose TSPY as a marker, because this gene has been mapped to a smallest region of the Y chromosome consistently present in females with gonadoblastoma, and TSPY has been proposed to be responsible for the origin of this tumour [18]. We attempted to analyse the various steps from the hypothesised development of some dysgerminomas from gonadoblastoma, considering why these neoplasias develop and progress.

## Results

### Immunohistochemistry pattern of expression in OGCTs and foetal ovaries

**Figure 1:**
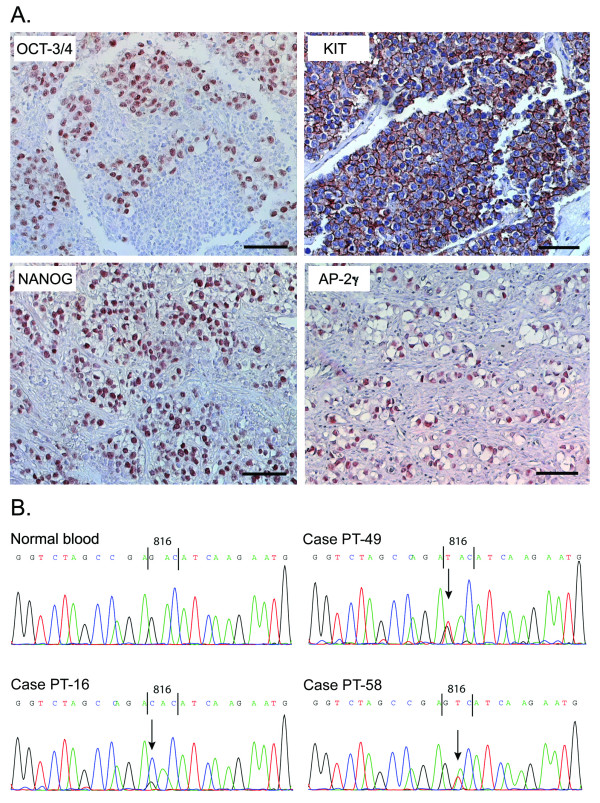
**Expression of stem cell related markers and *KIT *mutations in dygerminomas**. **A**. Immunohistochemical staining for OCT-3/4, KIT, NANOG, and AP-2γ in dysgerminomas. Scale bar = 25 μm. **B**. Examples of *KIT *mutation analysis, with control sequence from normal blood, and three of the mutated *KIT *(codon 816) sequences from dysgerminomas.

**Figure 2:**
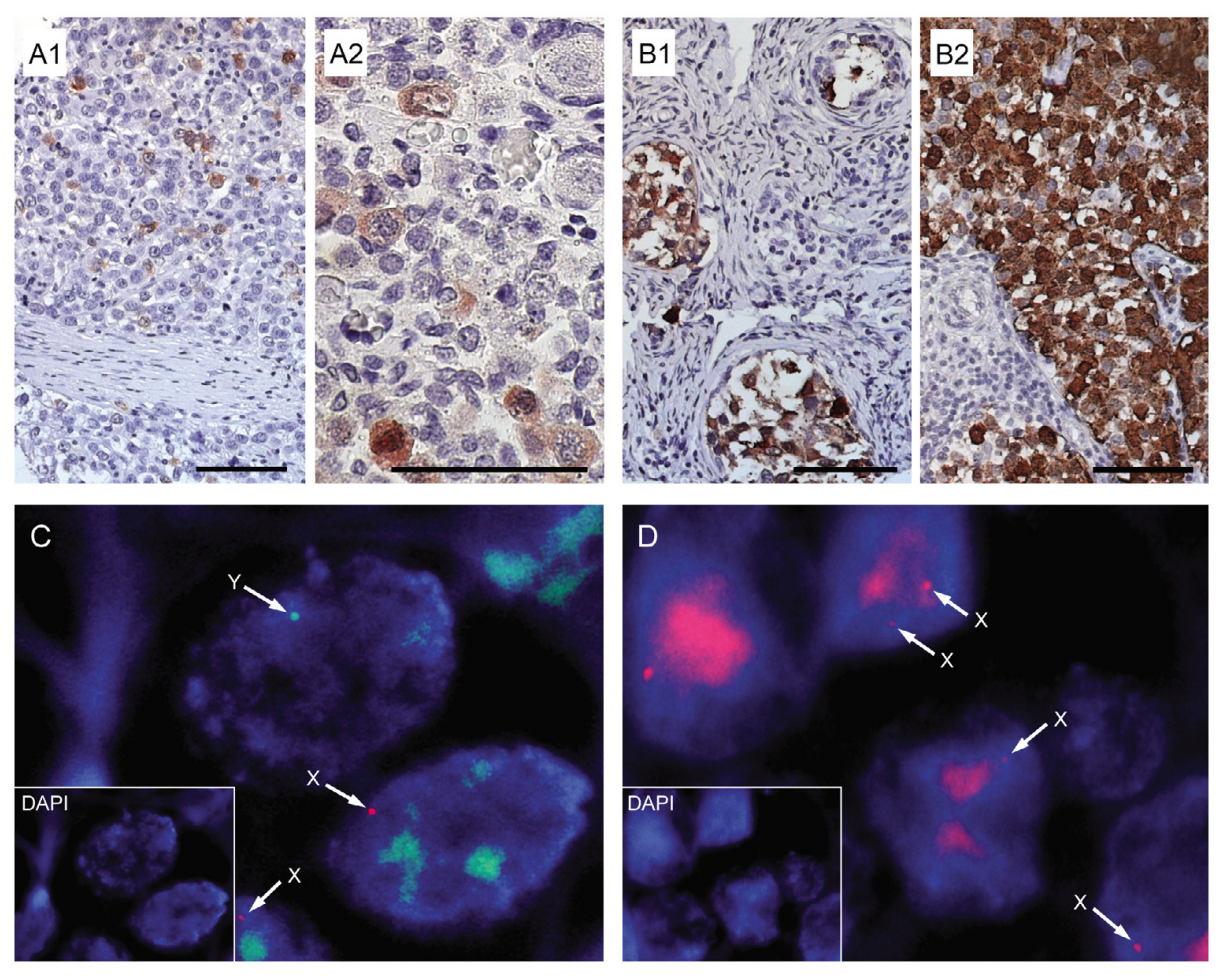
***In situ *expression pattern of germ cell-related markers (MAGE-A4 and TSPY) in OGCTs and sex centromere material in interphase nuclei from OGCTs**. MAGE-A4 in **A1**: Dysgerminoma and **A2**: In foetal ovary of GW 28 with strong expression in oogonia (lower left), and no expression in developing follicles (top right); TSPY in **B1**: Gonadoblastoma (Case PT-04) and **B2**: Dysgerminoma (Case PT-57). Scalebar = 25μm. Fluorescence *in situ *hybridisation of two different dysgerminomas with sex chromosome centromeres: **C**. Presence of X and Y chromosome material (Case PT-04) and **D**. Presence only of X chromosome material (Case PT-14). E-F: Inserts are control DAPI-only.

The expression pattern in OGCTs of a panel of markers for testicular GCTs is summarised in Table 1 and illustrated in Fig. 1 and 2. Placental alkaline phosphatase (PLAP), a classical marker of GCTs, was expressed in 100% of dysgerminomas, gonadoblastomas, embryonal carcinomas, and in 46% of yolk sac tumours. Expression of the stem cell-related markers OCT-3/4 and KIT were present in 80% of dysgerminomas, 75–100% of tumours containing gonadoblastoma and in two yolk sac tumours, whereas NANOG and AP-2γ were present in approximately half of the dysgerminomas and gonadoblastomas. All bilateral (n = 6) OGCTs were KIT positive. Overall the tumours containing dysgerminoma and/or gonadoblastoma showed expression of 0–4 stem cell markers (mean 2.5). Only one dysgerminoma and one dysgerminoma with gonadoblastoma did not express any of the stem cell markers. Bilateral cases expressed a mean of 3.4 stem cell markers, whereas unilateral cases expressed a mean of 2.1 markers. MAGE-A4 staining was present heterogeneously in 40% of dysgerminomas and in 13% of dysgerminomas with gonadoblastoma. No OGCTs showed expression of NY-ESO-1 (CTAG1B/LAGE) or AMH (anti-Müllerian hormone). As the OGCTs may develop in dysgenetic ovaries with some testicular differentiation, we examined the expression of AMH (anti-Müllerian hormone), a glycoprotein involved in involution of Müllerian ducts in the foetus leading to male differentiation [19], but did not detect expression in any OGCTs. Analysed cores of tumour tissues from mixed OGCTs did not differ in expression pattern from cores from pure OGCTs. Most of the markers expressed by dysgerminoma and gonadoblastoma were negative in non-dysgerminomas and none of the ovarian small cell carcinomas expressed any of the analysed markers. In foetal ovaries of all analysed ages, strong expression of MAGE-A4 was detected in the majority of oogonia (Fig. 2) and NY-ESO-1 in a subset of oogonia. However, neither of the two was expressed in developing follicles. NANOG and TSPY were not expressed in foetal ovary of GW 13–40.

**Table 1:** Protein expression of stem cell markers and others in OGCTs

**HISTOLOGY of core**	**N**	**N bilat**	**OCT-3/4**		**NANOG**		**KIT**		**AP-2γ**		**TSPY**		**MAGE-A4**		**NY-ESO-1**		**AMH**		**PLAP**		**HCG**		**AFP**	
**OVARIAN TISSUES**																								

**Foetal GW 13–18**	4		OCT-3/4 in foetal ovary: Ref [21]		Neg		KIT in foetal ovary: Ref [31]		AP-2γ in foetal ovary: Ref [27]		Neg		Oogonia++		Oogonia+/-		AMH in foetal ovary: Ref [19]		n.a.		n.a.		n.a.	

**Foetal GW 20–24**	4				Neg						Neg		Oogonia++		Oogonia+/-				n.a.		n.a.		n.a.	

**Foetal GW 34–40**	4				Neg						Neg		Oogonia++		Oogonia+/-				n.a.		n.a.		n.a.	

**DG**	**17**	**4**	**++/+-**	**80%**	**++**	**67%**	**++/+**	80%**	**++/+**	**33%**	**+-**	**13%**	**++/+-**	**40%**			**-**		**++/+-**	**100%**	**++**	**7%**	**++**	**7%**
**DG (w/GB)**^¤^	**8**	**4**	**++/+-**	**75%**	**++/+**	**38%**	**++**	88%**	**+/+-**	**38%**	**++/+**	**63%**	**+**	**13%**	**-**		**-**		**++/+-**	**100%**	**-**		**-**	
**GB**	**2**	**1**	**++/+**	**100%**	**+**	**50%**	**++/+**	100%**	**-**		**++**	**50%**	**-**		**-**		**-**		**++**	**100%**	**-**		**-**	
**EC**	**1**		**-**		**+**	**100%**	**-**		**-**		**-**		**-**		**-**		**-**		**++**	**100%**	**-**		**-**	
**YST**	**14**	**1**	**+**	**8%**	**+**	**15%**	**+/+-**	**15%**	**-**		**-**		**-**		**-**		**-**		**++/+-**	**46%**	**-**		**++/+-**	**77%**
**IT**	**17**		**-**		**-**		**-**		**-**		**-**		**-**		**-**		**-**		**-**		**-**		**+-**	**6%**
**CC**	**1**		**-**		**-**		**-**		**-**		**-**		**-**		**-**		**-**		**-**		**++**	**100%**	**-**	
**SCC**	8		-		-		-		-		-		-		-		-		-		-		-	

**NON-OVARIAN TISSUES**																								

**Adult normal testis**	3		-		+-	(unsp)	+	100%^†^	-		++	100%^||^	+	100%^||^	+	33%^††^	++	100%^‡^	-		-		-	
**CIS testis**	2		++	100%	++	100%	++	100%	++	100%	++	100%	-		-		-		n.a.		n.a.		n.a.	
**Seminoma testis**	2		++	100%	++	100%	++	100%	++	50%	++	100%	-		-		-		++	100%	-		-	
**EC/TER testis**	2		++	100%	++	100%	+	50%	++/+	100%	+	100%	-		-		-		++/+	100%	++	100%	+	50%
**YST testis**	1		++	100%	++	100%	-		-		-		-		-		-		+	100%	++	100%	++	100%
**Colon cancer**	2		-		-		-		-		-		-		-		-		-		-		-	
**Prostate cancer**	2		-		-		-		-		-		-		-		-		-		-		-	
**Normal brain**	1		-		-		-		-		-		-		-		-		-		-		-	
**Normal kidney**	1		-		-		-	(unsp)	-	(unsp)	+-	(unsp)	-		-		-		-		-		-	
**Normal liver**	2		-		-		-	(unsp)	-	(unsp)	-		-		-		-		-		-		-	

Staining intensity is marked by '++'/'+'/'+-'/'-' followed by the percentage of positive tissue samples. Staining intensity = '++': >50%, of tumour cells stained; '+': staining in approximately 20–50% of tumour cells; '+-': few cells stained; '-': no positive cells detected. For each tumour type the range of staining intensities are divided with '/'. Symbols = ^†^: staining in spermatocytes; ^||^: staining in spermatocytes and spermatogonia; ^††^: staining in spermatogonia; ^‡^: staining in pre-pubertal Sertoli cells; **: all bilateral DG or GB stained '++' with KIT; ^¤^: DG tissue from patients with diagnosis DG with GB. Abbreviations: OGCT = ovarian germ cell tumour; GW = gestational week; DG = dysgerminoma; GB = gonadoblastoma; EC = embryonal carcinoma; YST = yolk sac tumour; IT = immature teratoma; CC = choriocarcinoma; SCC = small cell carcinoma; CIS = carcinoma *in situ*; TER = teratoma; N = number; bilat = bilateral; n.a. = not analysed; ref = reference; unsp = unspecific.

### Analysis of tumour DNA for *KIT *gene mutations

**Figure 3:**
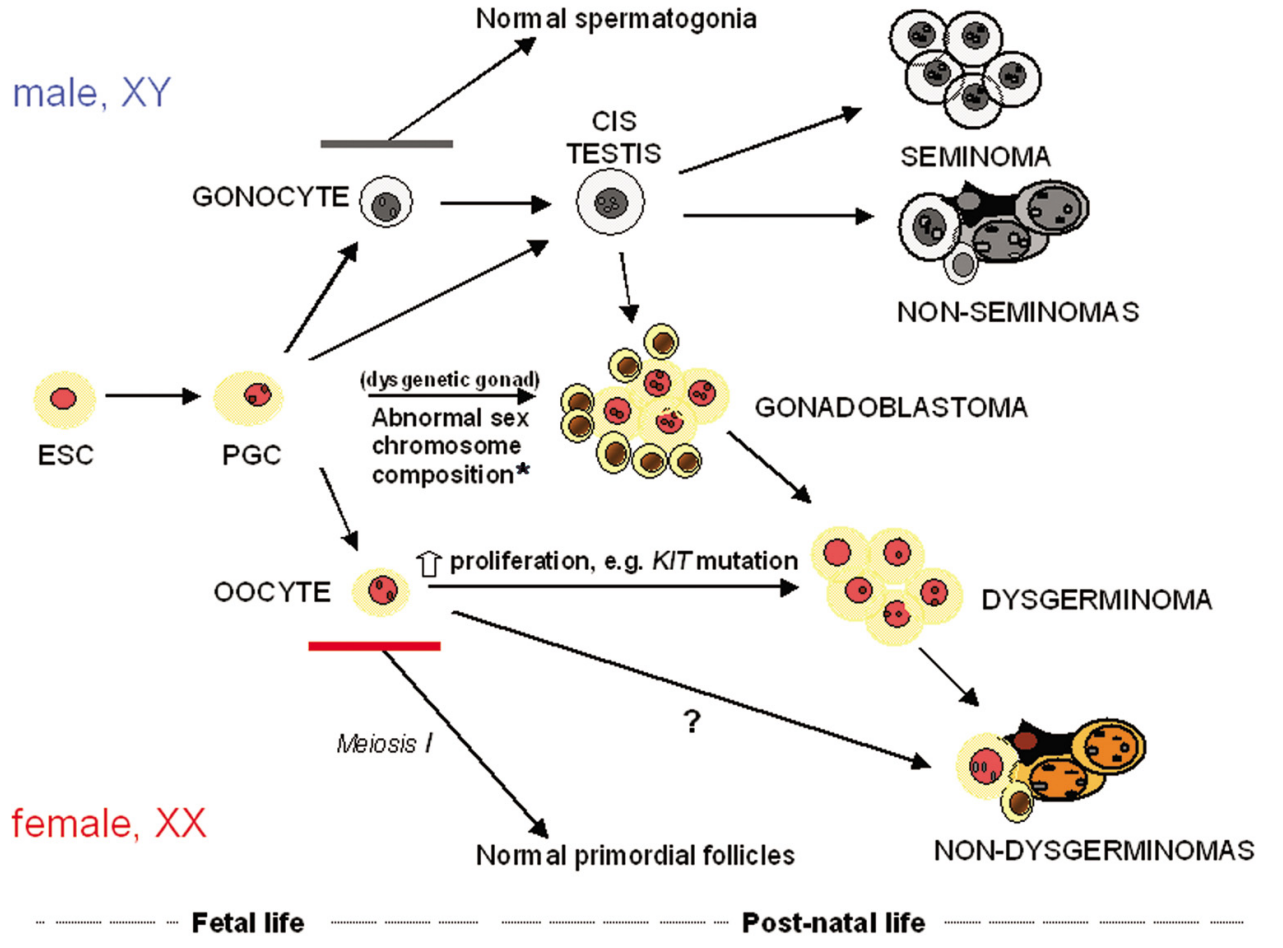
**A model for development of ovarian and testicular germ cell tumours**. * usually including Y chromosome material.

PCR amplification was performed on 52 tumour DNA samples from 43 patients (Table 2 and Fig. 3). *KIT *was mutated in codon 816 in 5/14 unilateral dysgerminomas, whereas all dysgerminomas from bilateral cases (n = 4) showed a wild type sequence, as did all OGCTs of other histotypes (n = 22). A mutation of G to C in the first base of the codon (GAC to CAC) was found once, whereas a G to T (GAC to TAC) was detected in two cases. The second base exchange GAC to GTC was also detected in two cases. The mutations in *KIT *were not linked to KIT protein overexpression, as 2/5 samples containing mutations were negative for KIT. On the other hand, all cores from bilateral cases were KIT positive by immunohistochemistry.

**Table 2:** DNA sequence status of *KIT *exon 17 and expression of KIT in OGCTs

**Patient ID**	**Histology of core**	**Bilateral**	**KIT protein expression**	**KIT mutation in codon 816, exon 17**
**PT-09**	DG (w/GB)		-	GAC to TAC, Asp to Tyr
**PT-16**	DG		+	GAC to TAC, Asp to Tyr
**PT-49**	DG		++	GAC to CAC, Asp to His
**PT-52**	DG		++	GAC to GTC, Asp to Val
**PT-58**	DG		-	GAC to GTC, Asp to Val
**PT-04**	DG	Bilateral	++	wt
	GB	Bilateral	++	wt
**PT-32**	DG	Bilateral	++	wt
	YST	Bilateral	-	wt
**PT-35**	DG (left ovary)	Bilateral	++	wt
	DG (right ovary)	Bilateral	++	wt
**PT-57**	GB	Bilateral	M	wt
	DG (left ovary)	Bilateral	++	wt
	DG (right ovary)	Bilateral	++	wt
**PT-03**	DG (w/GB)		++	wt
	EC		-	wt
	GB		-	wt
**PT-15**	DG		M	wt
**PT-19**	DG		-	wt
**PT-27, 39**	DG		++	wt
**PT-40**	DG (w/GB)		++	wt
**PT-48**	DG		+	wt
**PT-53**	CC		-	wt
	DG		+	wt
**PT-28**	DG		++	wt
	YST		-	wt
**PT-08**	YST		M	wt
**PT-20**	YST		+	wt
**PT-25,33,43,45,54,55**	YST		-	wt
**PT-06,10,18,23,31,34,44,46,47,51**	IT		-	wt
**PT-12, 22**	IT		M	wt
**PT-36, 37**	YST, IT		-	wt
**PT-11, 59, 56**	SCC		-	wt

Staining intensity = '++': >50%, of tumour cells stained; '+': staining in approximately 20–50% of tumour cells; '+-': few cells stained; '-': no positive cells detected. Abbreviations: DG = dysgerminoma; GB = gonadoblastoma; YST = yolk sac tumour; EC = embryonal carcinoma; CC = choriocarcinoma; IT = immature teratoma; OGCT = ovarian germ cell tumour; PT = patient; SCC = small cell carcinoma; M = missing; wt = wild type.

### Presence of Y-chromosome material in dysgerminoma/gonadoblastoma samples

TSPY protein was present in six tumour tissue cores from five patients, all harbouring dysgerminoma and/or gonadoblastoma elements. Four of five patients had bilateral neoplasia. Immunohistochemistry for TSPY was then repeated on whole tumour sections with identical results. Karyotypes of normal tissues (e.g. blood cells) of the OGCT patients were not available in the majority of cases, therefore we performed FISH with a centromeric probe for the Y chromosome on 27 sections containing dysgerminoma and/or gonadoblastoma, of which 19 were conclusive. Y-chromosome material was detected sections from five patients, and these were the patients harbouring TSPY-positive cells. In three other tissues staining was judged as unspecific, with no Y-chromosome material detected by FISH. Analysis on 12 non-dysgerminoma/gonadoblastoma samples (1 embryonal carcinoma, 7 yolk sac tumours, 4 immature teratomas) showed only X chromosome signal. Y-chromosome material was present in all control testicular GCTs (seminoma, yolk sac tumour, embryonal carcinoma). Examples are shown in Fig. 2.

## Discussion

In this study, we provide novel evidence for distinct, partly overlapping mechanisms acting in the pathogenesis of OGCTs. To our knowledge, this study is the first comprehensive analysis of an *in situ *protein expression profile of pluripotency genes and germ cell differentiation markers in these relatively rare malignant tumours. The expression of the transcription factors OCT-3/4 and NANOG were of particular interest, as they are key regulators of self-renewal and pluripotency of embryonic stem cells. In addition to the role in early embryonic development, these proteins are only present in PGCs, oogonia and gonocytes and in the testis only in malignant GCTs [20-22]. In the present study, OCT-3/4 expression was abundant in all gonadoblastomas and most dysgerminomas, in accordance with previously published data [23]. NANOG has not previously been evaluated in the ovary. Approximately half of the dysgerminomas and gonadoblastomas expressed NANOG, whereas tumours with somatic differentiation were largely negative. We did not detect NANOG expression in foetal ovaries, but cannot exclude expression in first trimester. Murine foetal ovary expresses Nanog in early foetal development with earlier downregulation than in male gonads [24]. Our results indicate that in analogy to the mouse model, NANOG could to be earlier down-regulated in human foetal ovary than testis, where gonocytes abundantly express NANOG until GW 20 [22].

The tissue specific adult stem cell-related factors AP-2γ and KIT are highly expressed in immature gonocytes, testicular carcinoma *in situ *(the pre-invasive stage of testicular GCTs) and in seminomas, and AP-2γ, but not KIT, is expressed in embryonal carcinoma [25-27]. AP-2γ expression was previously reported in ovarian carcinomas [28] and in 5/5 dysgerminomas [29]. In contrast to the male pattern, AP-2γ was only expressed in 33% of dysgerminomas and 38% of dysgerminomas containing gonadoblastoma. Higher oestrogen levels in the ovaries may in part explain the observed difference, as AP-2γ has been suggested involved in oestrogen signalling [27]. The role of AP-2γ in the gonads has not yet been elucidated but disruption of the gene in mice leads to complete loss of germ cells, probably because of failure of *KIT *induction, which is hypothesised to be a main target for AP-2γ[30].

The KIT/SCF system is of particular interest in regard to the origin of GCTs, due to a role in PGC proliferation and survival in the developing human gonad [9]. KIT expression was previously described in oogonia and oocytes [25,31,32] and in some OGCTs, predominantly dysgerminomas [33-35]. In accordance with these reports, we found KIT expressed in 80–100% of neoplastic cells in dysgerminomas and gonadoblastomas, and in 15% of yolk sac tumours. The high expression of KIT in dysgerminoma prompted us to investigate the possibility of a gene mutation. A gain-of-function mutation in the *KIT *gene was first reported in human GCTs, including one ovarian dysgerminoma/yolk sac tumour by Tian *et al*. [10] and later studies have shown *KIT *mutations in a varying proportion of phenotypically indistinguishable testicular seminomas [12,14,15,36], primary mediastinal seminomas [11] and intracranial germinomas, both in males and females [13,17]. Among 11 OGCTs previously analysed for *KIT *mutations two (one unilateral case – for the other such info was not given) had a GAC to CAC change in codon 816 [10,14,16]. We detected five *KIT *mutations in the same codon, but found three different base changes in five OGCTs (four pure dysgerminomas and one dysgerminoma with gonadoblastoma). Strikingly, all mutations were found in unilateral cases and none among four analysed bilateral cases. This could suggest that KIT mutations occur after completion of PGC migration to the gonadal ridges in females, and may be in contrast to what has been suggested for males, where some reports have shown *KIT *mutations more frequently in bilateral seminomas [12,15]. We hypothesise that activating *KIT *mutation(s) in early oogonia may stimulate their proliferation and delay meiotic entry, which in normal ovaries occurs gradually in foetal life, thus increasing the pool of immature germ cells.

It has been proposed that dysgerminoma may evolve from gonadoblastoma [37,38]. We analysed the stages of tumour development, from pure gonadoblastoma, through dysgerminoma with gonadoblastoma, to pure dysgerminoma, using the *TSPY *gene, a proposed GBY gene, as a marker. Besides gonadoblastoma, *TSPY *is expressed in male pre-meiotic germ cells and testicular GCTs that retain germ cell phenotype [18,39,40]. We found abundant TSPY in 5/7 cases of gonadoblastoma or dysgerminoma with gonadoblastoma, but not in pure dysgerminoma (n = 11). Consistent results were detected with TSPY protein and FISH Y-centromere analyses, suggesting that *TSPY *is included in the sub-region of the Y-chromosome involved. In one case of combined dysgerminoma/gonadoblastoma we detected a *KIT *mutation. Taken together, these results indicate that development of dysgerminoma with gonadoblastoma versus pure dysgerminoma may follow different molecular routes, with a role for one or more Y-chromosome genes in the development of gonadoblastoma in phenotypic females. Sex-chromosome alteration leads to gonadal dysgenesis and partial masculinisation in at least some cells of the developing female ovary. This may lead to a delay of the normal differentiation of germ cells in foetal ovaries and a failure of the onset of meiotic prophase, thus increasing the chance of mutational events (e.g. in *KIT*) and neoplastic transformation in the mitotically dividing germ cells, perhaps stimulated by pleiotrophic action of TSPY.

As the OGCTs may develop in dysgenetic ovaries with some testicular differentiation, we examined the expression of AMH, a glycoprotein involved in involution of Müllerian ducts in the foetus leading to male differentiation [19], but did not detect expression in any OGCTs. We furthermore examined MAGE-A4 and NY-ESO-1 [41], two representatives of the germ cell-specific "cancer/testis" gene family on the X-chromosome, which appear in gonocytes from around gestational week 17–18 at differentiation into pre-spermatogonia, and are expressed in adult spermatogonia and primary spermatocytes, as well as in some testicular tumours and a broad range of somatic cancers [42,43]. In accordance with previous studies [43,44] we detected expression of MAGE-A4 and NY-ESO-1 in foetal oogonia/oocytes of 13–40 weeks of gestational age. MAGE-A4 and NY-ESO-1 have been reported in ovarian epithelial and serous carcinomas [45], but this is the first study regarding OGCTs. We found a heterogeneous expression of MAGE-A4 but not NY-ESO-1 in dysgerminomas, analogous to the pattern in testicular seminomas.

In this study, we included also some small cell carcinomas of the hypercalcaemic type that occasionally develop in the ovaries of young patients. None of the applied markers were positive, and therefore it is unlikely that small cell carcinomas have a germ cell progenitor, as has been suggested [46,47]. With regard to the immature teratomas, they were also negative for all analysed markers, which may support the observation that this tumour, which is usually diploid and does not have the isochromosome (12p) imbalance, probably develops in a different manner than other malignant OGCTs [3,4]. No *KIT *mutations or presence of Y-chromosome material was detected in any of the small cell carcinomas or immature teratomas.

We detected many similarities, but also some notable differences in the protein expression profiles of ovarian and testicular GCTs, but nevertheless propose a comparable developmental model, as depicted in Fig. 3. Genetic alterations and biological mechanisms may be identical, with abnormal persistence of undifferentiated PGCs in both sexes, with the large difference in incidence reflecting the much lower number of susceptible cells in females at the time of puberty [48]. The proposed stepwise progression from gonadoblastoma to dysgerminoma in dysgenetic ovaries [38], and the similarities of gonadoblastoma and testicular carcinoma *in situ *[49], could indicate that gonadoblastoma – analogous to carcinoma *in situ *in the testes – is a precursor stage, which probably only occurs in phenotypic females with Y-chromosome material. As the stem cell-related markers were abundantly expressed, our hypothesis is that in 46, XX females, the OGCTs probably are derived from PGCs or oogonia. We speculate that despite the lack of the Y-chromosome material, some degree of gonadal dysgenesis leading to a delay in the differentiation of PGC/oogonia and initiation of gonadoblastoma-like lesions, may be due to changes in hormonal environment, perhaps caused by endocrine disrupters or by lifestyle changes, e.g. obesity. However, the low incidence of OGCTs complicates establishment of epidemiological associations.

## Conclusion

We provide in this study new evidence for two partially overlapping mechanisms that may contribute to the pathogenesis of ovarian germ cell neoplasms. One mechanism is related to the presence of Y-chromosome material and ensuing gonadal dysgenesis. The other mechanism is primarily genetic and involves spontaneous mutation(s) in the *KIT *gene, leading to increased survival and proliferation of undifferentiated oogonia. We expect this mechanism not to be limited to *KIT*, but demonstrate that mutations in *KIT *are responsible for a sizeable number (5/17) of cases containing dysgerminoma. The neoplastic transformation must occur before oocytes enter meiosis, most likely after the migration to the gonadal ridges, as the mutations were detected in unilateral tumours.

## Methods

### Tissue samples

The samples originated from patients diagnosed between 1983–2001, who were admitted to 18 Norwegian hospitals for surgery and subsequently referred to the Norwegian Radium Hospital for adjuvant treatment. The tissue microarray (TMA) consisted of 60 cores from tumours of 50 OGCTs patients as described in Table 3. The TMA also contained tissue cores of ovarian small cell carcinoma (n = 8) and various non-ovarian normal and neoplastic tissues, see Table 1. For studies of the ontogeny of expression of selected proteins, we used normal foetal ovarian specimens, which were obtained after induced or spontaneous abortions and stillbirths, mainly due to placental or maternal problems (n = 12, from Rigshospitalet, Copenhagen University Hospital). Developmental age was calculated according to menstrual bleeding and foetal foot size [25]. A representative series of paraffin-embedded testicular GCT samples from the tissue bank at Rigshospitalet was used for positive controls. The Norwegian ethical guidelines were followed and the biobank is registered at the Norwegian Institute of Public Health. The Regional Committee for Medical Research Ethics in Denmark approved the use of Danish tissue samples.

**Table 3:** Overview of the sample content of the Ovarian Germ Cell tumour – Tissue Microarray

**Diagnosis of tumour(s)**		**No. CORES**	**No. PATIENTS**	**Histology of analysed component(s)**
**Pure DG**	- Unilateral - Bilateral	10 3	10 2	DG 1 case one side; 1 case both sides
**DG/GB**	- Unilateral - Bilateral	3 6	3 3	2 cases DG and GB; 1case only DG
**Pure YST**	- Unilateral	7	7	YST
**Pure IT**	- Unilateral	15	15	IT
**Mixed OGCT: DG/YST**	- Unilateral - Bilateral	2 2	1 1	DG and YST DG and YST
**Mixed OGCT: YST/EC**	- Unilateral	1	1	YST
**Mixed OGCT: DG/EC/GB**	- Unilateral	3	1	DG, EC, and GB
**Mixed OGCT: YST/IT**	- Unilateral	6	5	3 cases only YST; 1 case only IT; 1 case YST and IT
**Mixed OGCT: DG/CC**	- Unilateral	2	1	DG and CC
**SCC**	- Unilateral	8	8	SCC

The tissue microarray (TMA) contained of 60 cores obtained from OGCTs of 50 patients and 8 small cell carcinomas from 8 patients. Six patients exhibited bilateral neoplasia. H&E stained sections from several blocks were re-evaluated by a pathologist (V.M.A.), classified according to the WHO recommendations [1], and the most optimal tissue blocks were chosen. The TMA was constructed by transferring core biopsies of 0.6 μm in diameter from each donor block into an acceptor block (Beecher Instruments, Sun Prairie, USA). Abbreviations: GCT = germ cell tumour; No. = number; DG = dysgerminoma; GB = gonadoblastoma; YST = yolk sac tumour; IT = immature teratoma; EC = embryonal carcinoma; CC = choriocarcinoma; SCC = small cell carcinoma.

### Immunohistochemistry

The protein expressions were analysed by immunohistochemistry on 4 μm formalin-fixed, paraffin-embedded sections. The following antibodies were used: OCT-3/4 (C-10, sc-5279) and AP-2γ (6E4/4, sc-12762) from Santa Cruz Biotechnology, CA); NANOG (14-5769) from eBioscience, San Diego, CA; TSPY from Y.-F.C. Lau, VA Medical Center, University of CA, San Francisco; MAGE-A4 and NY-ESO-1 from G. Spagnoli, Ludvig Institute for Cancer Research, Switzerland; AMH from R.L. Cate, Biogen, MA; HCG (A0023), KIT (CD117, A4502), and AFP (A0008) from DakoCytomation, Glostrup, Denmark; PLAP (NCL-PLAP-8A9) from NovoCastra, Newcastle, UK. A previously published standard indirect peroxidase method was applied with small adjustments [19]. Briefly, most of the dewaxed and rehydrated sections were heated in a microwave oven in one of the following buffers: TEG-buffer = TRIS 1.21 g/L, EGTA 0.19 g/L, pH 9.0 (OCT-3/4, KIT, TSPY); Tris EDTA-buffer = 1.2 g/L TRIS, 0.37 g/L EDTA (HCG); 5% Urea-buffer, pH 8.5 (AP-2γ, AMH); Citrate-buffer = 10 mmol/L, pH 6.0 (NANOG, MAGE-A4); Target Retrieval Solution (DakoCytomation), low pH (PLAP) and high pH (AFP)). Sections were then incubated with 1.5% H_2_O_2_, followed by diluted non-immune goat serum or human serum. Incubation with the diluted primary antibody was overnight at 4°C (dilution OCT-3/4 1:300; NANOG 1:80; KIT 1:400; AP-2γ 1:50; TSPY 1:7000; MAGE-A4 1:200; NY-ESO-1 1:50; AMH 1:150; HCG 1:4000; PLAP 1:20; AFP 1:800). A serial section was incubated with dilution buffer for each investigation as negative control. Subsequently, a secondary biotinylated link antibody was applied, followed by the horseradish peroxidase-streptavidin complex (all reagents from Zymed, S. San Francisco, CA). Visualization was with Envision+ (DakoCytomation, AFP, HCG, PLAP) or aminoethyl carbazole substrate (Zymed, all other antibodies) and counterstaining with Mayer's haematoxylin.

To define the pattern of expression of the analysed proteins and to control for specificity of all antibodies, we analysed expression patterns in various normal tissues, testicular GCTs, and non-gonadal malignancies and results were in accordance with previously reported expression patterns (Table 1).

Evaluation was performed independently by V.M.A and C.E.H.H. by a semi-quantitative score (Table 1) after transfer of the TMA images to a computer by a high-resolution camera (Sony DFW-SX900 CCD Digital Color Camera) using an in-house developed software (Dept. Medical Informatics, Rikshospitalet-Radiumhospitalet Medical Centre, Oslo, Norway) enabling examination of several cores simultaneously. When discrepancy arose cores were re-evaluated and a consensus reached.

### *KIT *mutation analysis, codon 816

Mutation analysis of the *KIT *gene was focused on exon 17, in which the most commonly mutated codon 816 is located. Analysis was performed by PCR and direct sequencing. Genomic DNA was extracted from formalin-fixed, paraffin-embedded tissue, as described in [4] and amplified with the primers: *KIT *exon 17-F: 5'-TTTCTCCTCCAACCTAATAG-3', *KIT *exon 17-R: 5'-CCTTTGCAGGACTGTCAAGC-3' [50]. The PCR reaction contained HotStarTaq DNA Polymerase (Qiagen, Hilden, Germany) and 10× HotStar buffer with 15 mM MgCl_2_, with amount of template between 25–200 ng in a total reaction volume of 25 μl. Amplification was performed by: 95°C for 15 min of initial heat denaturation followed by 35–40 cycles of 94°C for 30 sec, 48–52°C for 30 sec, and 72°C for 30 sec, followed by 72°C for 8 min. The PCR products were purified with ExoSAP-IT containing Exonuclease 1 and Shrimp Alkaline Phosphatase (USB, Cleveland, Ohio) and subsequently directly sequenced with the BigDye Terminator v1.1 Cycle Sequencing Kit (Applied Biosystems, Foster City, CA). Briefly; 1 μl ready reaction mix, 1 μl PCR-product, 1,5 pmol of sequence primer, 1,5 μl BigDye buffer, and MilliQ-water up to a total reaction volume of 10 μl. Forward and reverse sequencing was performed with an Applied Biosystems 3730 DNA Analyzer with independent analysis by two researchers.

### Fluorescence *in situ *hybridisation (FISH)

FISH was performed to analyse the presence of X and Y-chromosome material. Preparation of the sections was similar to that described above for immunohistochemistry. Sections were deparaffinised, rehydrated and pre-treated with proteinase K solution/PBS (20 μg/ml, Invitrogen, Carlsbad, CA). Dehydration was applied before denaturation of slides together with the probes specific for centromeres of the X-chromosome (Texas red labelled) and the Y-chromosome (FITC labelled, green fluorescence) (probes from DakoCytomation). Sections were hybridised for 2 h, washed, dehydrated and mounted in Antifade with DAPI II (125 ng/ml, Vysis Inc., IL), followed by examination using an epifluorescence microscope (Leica, Wetzlar, Germany) with 100× magnification and the CytoVysion software (Applied Imaging, Newcastle, UK).

## Declaration of competing interests

The author(s) declare that they have no competing interests.

## Authors' contributions

C.E.H.H. performed and co-analysed most immunohistochemical stainings, supervised FISH analysis, performed part of the data analysis and drafted the manuscript. S.M.K. coordinated and designed the tissue microarray, performed part of the mutation analysis and data analysis, and contributed to writing of the manuscript. V.M.A. performed pathology evaluation of the tumours and guided tissue selection for microarray preparation, co-analysed immunohistochemical stainings, and contributed to writing of the manuscript. J.K. was responsible for inclusion of the clinical data and performed the clinical follow-up of many of the patients. E.R.M. supervised immunohistochemical stainings contributed to the planning, data analysis, and writing of the manuscript. R.A.L. initiated the project, supervised KIT mutation analysis, contributed to the planning, data analysis, and writing of the manuscript. All authors read and approved the final manuscript.
